# Optimal Irradiation Strategy to Induce Male Sterility in Cotton Mealybug, *Phenacoccus solenopsis* Tinsley (Hemiptera: Pseudococcidae)

**DOI:** 10.3390/plants14060912

**Published:** 2025-03-14

**Authors:** Wanying Dong, Yang Lei, Chaogang Liu, Farman Ullah, Jun Huang, Zhongshi Zhou, Yaobin Lu

**Affiliations:** 1 State Key Laboratory for Managing Biotic and Chemical Threats to the Quality and Safety of Agro-Products, Key Laboratory of Biotechnology in Plant Protection of MOA of China and Zhejiang Province, Institute of Plant Protection and Microbiology, Zhejiang Academy of Agricultural Sciences, Hangzhou 310021, China; dongwy@zaas.ac.cn (W.D.); ly538253@163.com (Y.L.); farmanullah787@gmail.com (F.U.); junhuang1981@aliyun.com (J.H.); 2 College of Advanced Agricultural Sciences, Zhejiang A&F University, Hangzhou 311300, China; 3 Institute of Crops and Nuclear Technology Utilization, Zhejiang Academy of Agricultural Sciences, Hangzhou 310021, China; liucg@zaas.ac.cn; 4 State Key Laboratory for Biology of Plant Diseases and Insect Pests, Institute of Plant Protection, Chinese Academy of Agricultural Sciences, Beijing 100193, China; 5 National Nanfan Research Institute, Chinese Academy of Agricultural Sciences, Sanya 572019, China; 6 Institute of Bio-Interaction, Xianghu Laboratory, Hangzhou 311231, China

**Keywords:** *Phenacoccus solenopsis* Tinsley, sterile insect technique, γ-ray, optimal dose, radiation biology, mating competitiveness

## Abstract

*Phenacoccus solenopsis* Tinsley is a highly invasive pest threatening global cotton production and numerous cultivated crops. The sterile insect technique (SIT), based on γ-ray irradiation, offers a sustainable and eco-friendly alternative to chemical controls for managing pests. This study aimed to determine the optimal developmental stage and radiation dose to induce sterility in *P. solenopsis*. Male pupae aged 5 days demonstrated the highest tolerance to irradiation among all tested age groups. These 5-day-old male pupae were irradiated with 20, 40, 60, and 100 Gy and mated with unirradiated females, and the effects on mating rate, oviposition stage, egg number, egg hatchability, male adult lifespan, and female sex ratio were assessed. Sterility was effectively induced by 60 Gy in males without compromising their mating competitiveness in the parental (F_0_) generation. Moreover, sterility traits were inherited by descendants, leading to a progressive decline in population size across the F_1_ and F_2_ generations. Therefore, a 60 Gy dose was identified as optimal for suppressing *P. solenopsis* in field settings. These findings establish a theoretical foundation for implementing SIT as a regional pest management strategy for *P. solenopsis*.

## 1. Introduction

The cotton mealybug, *Phenacoccus solenopsis* Tinsley (Hemiptera: Pseudococcidae), originating in North America, was first identified as a damaging pest to cotton in the United States in 1991 [1]. It has since spread to numerous countries, including Mexico, Brazil, Chile, and Australia [2], and is now distributed across all continents except Antarctica, with over 200 recorded host plant species [3]. The nymphs and adult females of *P. solenopsis* harm plants directly by feeding on sap from tender tissues, leading to flower and fruit drop, stunted growth, yellowing, twisting, wrinkling of leaves, and ultimately defoliation. Additionally, the honeydew excreted by these pests promotes sooty mold growth, inhibiting photosynthesis and compounding the damage, which can result in widespread plant mortality in severe cases [4]. Currently, *P. solenopsis* poses significant threats to cotton and other cultivated crops in countries such as India and Pakistan [5].

In China, *P. solenopsis* was first discovered in Guangdong Province in June 2008 [6]. By July 2024, it had spread to 130 counties across 15 provinces and was designated as a national agricultural and forestry quarantine pest (http://www.moa.gov.cn/govpublic/ZZYGLS/202409/t20240902_6461580.htm) (accessed on 3 December 2024). Due to its small size, concealed nature, and ability to spread over long distances through human activity, *P. solenopsis* poses a significant threat to China’s cotton industry if it establishes colonies in major cotton-growing regions [7]. Current control measures rely heavily on chemical pesticides. However, the pest develops resistance rapidly, and its protective wax layer limits pesticide penetration, significantly reducing efficacy [4,7,8]. Furthermore, extensive pesticide use adversely impacts the agricultural environment and reduces the safety of agricultural products. Therefore, environmentally friendly and sustainable pest management strategies for *P. solenopsis* are urgently needed.

Insect radiation sterilization technology offers an eco-friendly alternative to chemical control. This physical method involves exposing target insects to high-energy rays or neutron flux, causing chromosomal damage and genetic disruptions in reproductive cells, resulting in asymmetric combinations and dominant lethality and sterility in current and subsequent generations [9,10]. Irradiated males mate with wild females, and the suppression effect is evaluated based on the degree to which their offspring (F_1_ generation) exhibit reduced reproductive capacity, a phenomenon known as F_1_ sterility. If the F_1_ generation is completely sterile, the applied dose is referred to as the complete sterility dose, while partial sterility in F_1_ is associated with a sub-sterility dose. Sub-sterility doses are particularly effective for broader pest control, as they minimize non-target effects on beneficial organisms and the environment [11]. Databases such as the International Database on Insect Disinfestation and Sterilization (IDIDAS) provide information on sterilization doses for various pests (https://nucleus.iaea.org/sites/naipc/ididas/Pages/Browse-IDIDAS.aspx) (accessed on 3 December 2024).

Research on radiation sterilization has primarily focused on Lepidoptera [12,13,14,15] and Diptera pests [16,17,18,19], with limited studies on Hemiptera mealybugs [20,21,22]. Existing research on *P. solenopsis* mainly focuses on quarantine-related irradiation rather than sterilization for pest management. For example, Huang et al. (2014) demonstrated that 200 Gy of ^137^Cs-γ-ray irradiation could serve as a quarantine treatment, while 100 Gy was sufficient to eliminate populations within two generations [23]. Similarly, Seth et al. used 500 Gy of ^60^Co-γ-ray irradiation on 11–12-day-old gravid females to completely prevent F_1_ egg hatch and 200 Gy to prevent first-instar nymphs from reaching the second instar [24]. However, the optimal age and sub-sterility dose for radiation-based sterilization in *P. solenopsis* remain unclear.

Several mealybugs and scale insects have asexual reproduction by parthenogenesis [25,26,27]. For *P. solenopsis*, at the beginning, both sexual and parthenogenetic reproductions were described, suggesting the parthenogenesis as the most common [27,28]. More recently, studies carried out by Huang et al. demonstrated that sexual reproduction is much more predominant in this species [29]. *Phenacoccus solenopsis* exhibits sexual dimorphism, with females undergoing incomplete metamorphosis from eggs through three nymphal stages to adulthood. Males, however, develop from eggs through two nymphal stages and additional prepupal and pupal stages before becoming adults [30]. These pupal stages and the flying ability of male adults provide a unique opportunity to develop sterile male strains using irradiation technology.

This study evaluated the impact of ^137^Cs γ-ray irradiation on the growth and reproductive capacity of *P. solenopsis*. Five radiation doses (20, 40, 60, 100, and 200 Gy) were applied to determine the optimal developmental stage and dose for inducing sterility. These results provide a scientific basis for implementing radiation sterilization as a sustainable pest management strategy for *P. solenopsis*.

## 2. Results

### 2.1. Optimal Day Age for P. solenopsis Sterility

**Figure 1:**
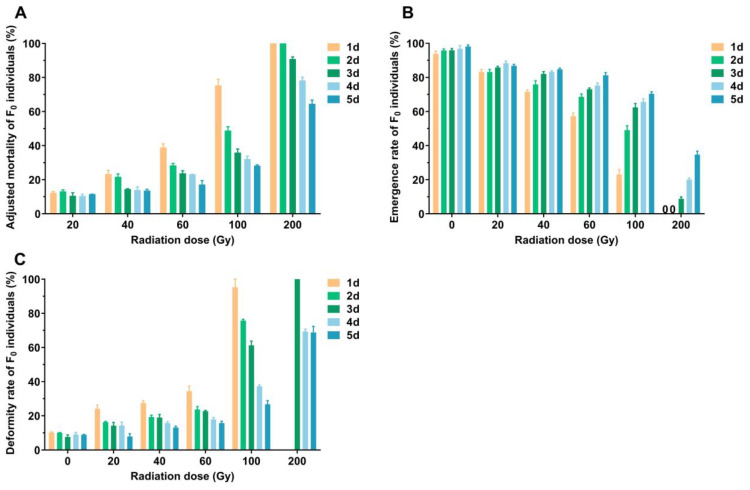
Effects of different doses of γ-ray irradiation on *Phenacoccus solenopsis* 1–5-day-old male (**A**) pupal adjusted mortality, (**B**) emergence rate, and (**C**) deformity rate. The data in the figure is the mean ± standard error.

γ-ray radiation at various doses significantly affected the survival and developmental parameters of 1*–*5-day-old male pupae of *P. solenopsis*. More specifically, the adjusted mortality rates of 1*–*5-day-old male pupae were significantly affected by radiation doses, age, and the interactions of radiation dose × age (*F*_4,75_ = 1782.452, *p* < 0.001; *F*_4,75_ = 199.454, *p* < 0.001; *F*_16,75_ = 35.047, *p* < 0.001, Table 1, Figure 1A). Across radiation doses ranging from 20 to 200 Gy, adjusted mortality rates increased with higher radiation doses for all age groups. At 200 Gy radiation, 1- and 2-day-old pupae reached a 100% adjusted mortality rate. Within the same radiation dose, older pupae exhibited lower adjusted mortality rates, with 5-day-old pupae consistently showing the lowest adjusted mortality at doses > 40 Gy.

The ED_50_ and ED_99_ values for pupae of varying ages were calculated according to mortality information (Table 2). The χ^2^ values for the Probit analysis were non-significant (*p* > 0.05), indicating a good fit of the model to the observed data. ED_50_ ranged from 75.23 Gy for 1-day-old pupae to 172.84 Gy for 5-day-old, while ED_99_ ranged from 206.54 Gy to 752.34 Gy. These results indicate that 1-day-old pupae were most vulnerable to γ-ray irradiation, while 5-day-old pupae were the most resistant.

The emergence rates of 1*–*5-day-old male pupae were significantly affected by radiation doses, age, and the interactions of radiation dose × age (*F*_5,90_ = 1131.385, *p* < 0.001; *F*_4,90_ = 125.998, *p* < 0.001; *F*_20,90_ = 16.664, *p* < 0.001, Table 1, Figure 1B). Across radiation doses ranging from 20 to 200 Gy, emergence rates decreased with increasing radiation doses across all age groups, with the lowest emergence rates observed at 200 Gy. At this dose, emergence rates for 1- and 2-day-old pupae dropped to 0, indicating no healthy male adults emerged. At the same dose, emergence rates increased with pupal age, with 5-day-old pupae showing the highest emergence rates at doses > 40 Gy.

The deformity rates of 1*–*5-day-old male pupae were significantly affected by radiation doses, age, and the interactions of radiation dose × age (*F*_5,84_ = 509.003, *p* < 0.001; *F*_4,84_ = 95.863, *p* < 0.001; *F*_18,84_ = 24.591, *p* < 0.001, Table 1, Figure 1C). Across radiation doses ranging from 20 to 200 Gy, deformity rates increased with higher radiation doses for all age groups. At 200 Gy, deformity rates peaked for 3- to 5-day-old pupae, with 3-day-old pupae reaching a deformity rate of 100%. Older pupae exhibited lower deformity rates at the same radiation dose. For 5-day-old pupae irradiated at doses > 20 Gy, deformity rates were consistently the lowest.

Based on these results, 5-day-old male pupae exhibited lower adjusted mortality, higher ED_50_ and ED_99_ values, higher emergence rates, and lower deformity rates compared to younger pupae. These findings suggest that 5-day-old male pupae were more resistant to irradiation and are suitable for subsequent experiments.

### 2.2. Preliminary Selection of Optimal Radiation Dose for P. solenopsis Sterility

**Figure 2:**
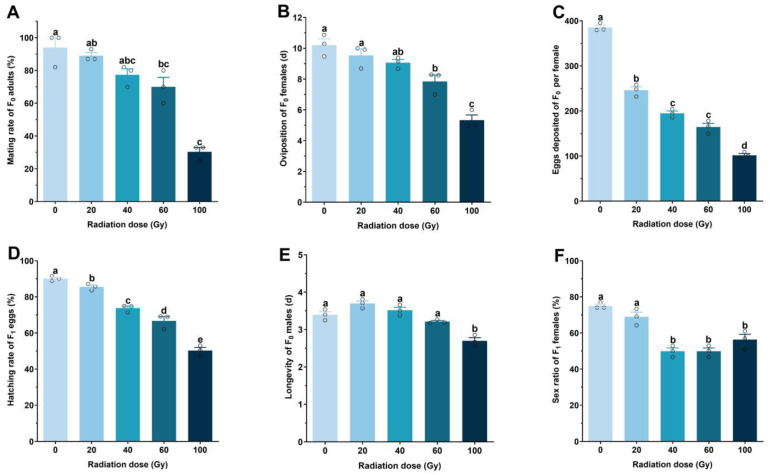
Effect of different doses of mean (± standard error) (**A**) mating rate, (**B**) oviposition, (**C**) eggs deposited per female paired with F_0_ irradiated males, (**D**) hatching rate of F_1_ eggs, (**E**) longevity of F_0_ males, and (**F**) sex ratio of females in F_1_ produced by couples of irradiated males and unirradiated females of *Phenacoccus solenopsis*. Different letters mean significant differences among different doses (*p* < 0.05). The circles on the bars represent the mean values for each of the three replicate groups.

Gamma irradiation significantly affected the mating rate of *P. solenopsis* males (Wald = 19.272, *p* < 0.001). Compared to the control (0 Gy), mating rates decreased significantly at doses of 60 and 100 Gy (Figure 2A). Females paired with males irradiated at 60 and 100 Gy exhibited shorter oviposition stages than those paired with unirradiated males (Wald = 31.557, *p* < 0.001; Figure 2B).

Egg production was significantly reduced in females mated with irradiated males. Egg numbers began to decline at 20 Gy and continued to decrease at 40 and 60 Gy, with no significant difference between 40 (194.79 eggs) and 60 Gy (164.51 eggs). At 100 Gy, egg production dropped further to 101.58 eggs, a significant reduction compared to 60 Gy (Wald = 857.831, *p* < 0.001; Figure 2C).

Egg-hatching rates declined with increasing radiation doses. At doses ≥ 20 Gy, hatching rates ranged from 50.28% to 85.49%, significantly lower than the control group’s rate of 90.09% (Wald = 409.816, *p* < 0.001; Figure 2D).

Male longevity was unaffected by doses < 60 Gy compared to controls (average longevity: 3.40 days). At doses between 20 and 60 Gy, average longevities ranged from 3.21 to 3.70 days. However, at 100 Gy, male longevity was significantly reduced (Wald = 7.942, *p* < 0.01; Figure 2E).

The female sex ratio did not significantly differ at 20 Gy (68.95%) but decreased significantly at doses > 40 Gy compared to controls (Wald = 7.414, *p* < 0.01; Figure 2F).

Based on these findings, irradiation at 60 Gy effectively induced partial sterility in males without affecting their lifespan, making it the optimal dose for subsequent experiments.

### 2.3. Effect of 60 Gy Gamma Radiation on Mating Competitiveness of Irradiated F_0_ Males

In treatments without irradiated males (UM, unirradiated male; IM, irradiated male; UF, unirradiated female; UM:IM:UF = 1:0:1), with only irradiated males (UM:IM:UF = 0:1:1), and with both irradiated and unirradiated males (UM:IM:UF = 1:1:1), the total number of eggs laid was 228.84 ± 3.35, 200.68 ± 4.21, and 211.53 ± 1.94, respectively. Egg hatching rates were 92.08 ± 1.18, 49.89 ± 3.04, and 63.05 ± 1.74, respectively. In the treatment with irradiated and unirradiated males, total egg numbers and hatching rates decreased by 7.56% and 31.53%, respectively, compared to the control treatment without irradiated males. The calculated competitiveness value was 2.21, indicating that irradiated males retained superior mating competitiveness over unirradiated males (Table 3).

### 2.4. Effect of 60 Gy Gamma Radiation on Descendants (F_1_ and F_2_) of P. solenopsis Irradiated F_0_ Males

**Figure 3:**
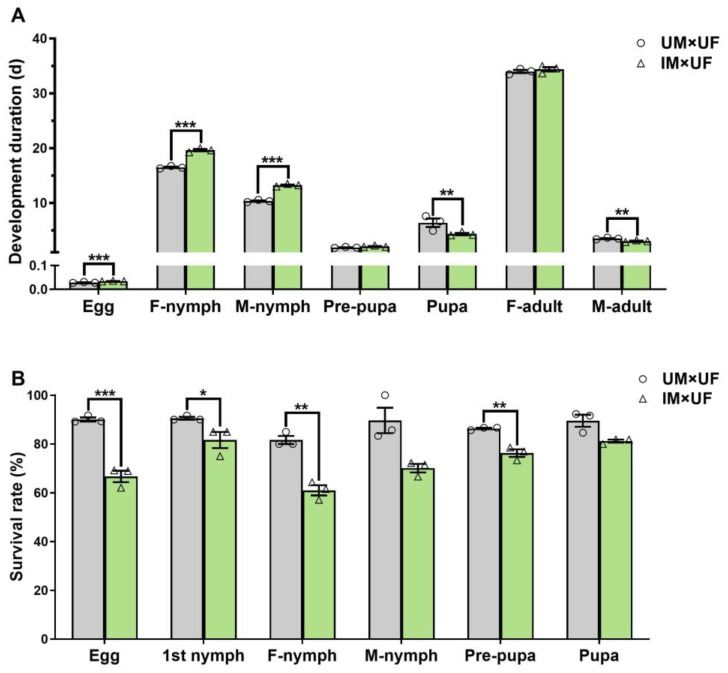
Effect of 60 Gy of gamma rays on (**A**) the development duration and (**B**) survival rate of the F_1_ generation. Asterisks indicate significant differences (* *p* < 0.05, ** *p* < 0.01, *** *p* < 0.001), independent *t*-test. Error bars represent standard error. The white line represents that the Y-axis is divided into two segments, with the bottom ranging from 0 to 0.1 and the top ranging from 1 to 40. UM, unirradiated male; IM, irradiated male; UF, unirradiated female; F, female; M, male.

The development duration of F_1_ progeny from male pupae irradiated with 60 Gy γ-rays exhibited significant changes (Figure 3A). F_1_ eggs, female nymphs, and male nymphs experienced prolonged development compared to controls. Specifically, the developmental durations of F_1_ eggs (0.034 days), female nymphs (19.63 days), and male nymphs (13.25 days) exceeded those of the control group (0.029, 16.50, and 10.36 days, respectively). In contrast, the developmental durations for F_1_ pupae (4.36 days) and male adults (3.05 days) were shorter than those of the controls (6.39 and 3.53 days, respectively). The developmental durations for F_1_ pre-pupae (2.08 days) and female adults (34.00 days) did not show significant differences compared to controls.

Gamma irradiation at 60 Gy significantly reduced the survival rates of F_1_ eggs, first-instar nymphs, female nymphs, and pre-pupae (Figure 3B). The survival rates of F_1_ eggs, first-instar nymphs, female nymphs, and pre-pupae decreased from 90.09%, 90.56%, 81.67%, and 86.35% in the control group to 66.71%, 81.67%, 61.00%, and 76.28%, respectively. No significant differences in survival rates were observed for F_1_ male nymphs (70.11%) or pupae (81.21%) compared to the control group.

**Figure 4:**
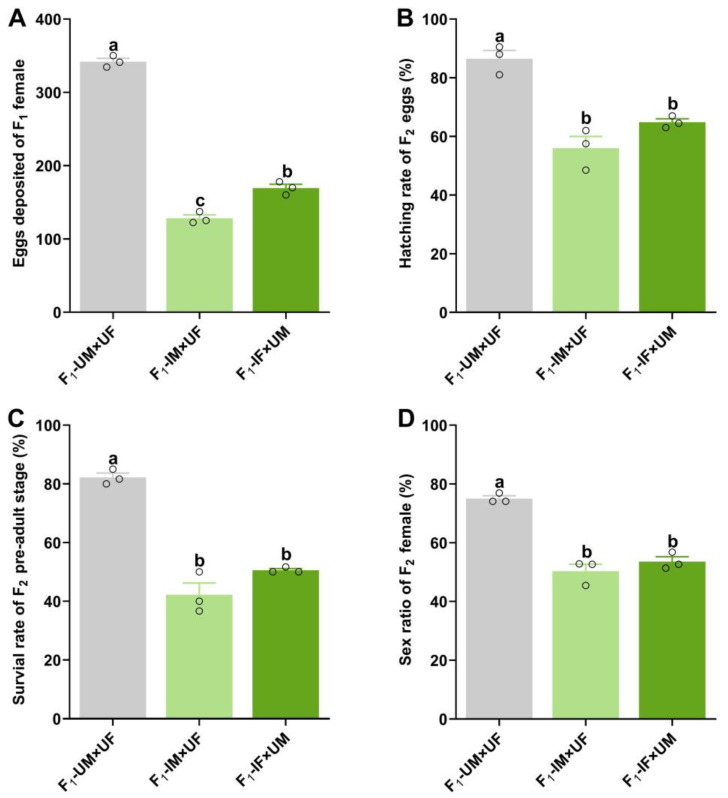
Effect of 60 Gy of gamma rays on F_1_ and F_2_ progeny. (**A**) Number of eggs, (**B**) hatching rate of F_2_ eggs, (**C**) survival rate of the F_2_ pre-adult stage, and (**D**) the sex ratio of females in the F_2_ generation from couples of unirradiated male and female adults (F_1_-UM + UF), F_1_ males from irradiated parental males and unirradiated female adults (F_1_-IM + UF), and F_1_ females from irradiated parental males and unirradiated male adults (F_1_-IF + UM). Different letters represent significant differences at the 5% level among different types of couples. Error bars represent standard error. The circles on the bars represent the mean values for each of the three replicate groups.

The fecundity of F_1_ progeny was markedly reduced when F_0_ males were irradiated with 60 Gy γ-rays (Figure 4A). The number of eggs laid by unirradiated females paired with F_1_ male progeny (F_1_-IM + UF) and F_1_ female progeny paired with unirradiated males (F_1_-IF + UM) was reduced by 62.48% and 50.46%, respectively, compared to unirradiated female/male pairs (F_1_-UM + UF) (Wald = 59.359, *p* < 0.001).

The radiation-induced effects extended to F_2_ progeny (Figure 4B–D). A significant reduction was observed in the hatching rate of F_2_ eggs (Wald = 115.817, *p* < 0.001), the survival rate of F_2_ pre-adult stages (Wald = 50.369, *p* < 0.001), and the sex ratio of F_2_ females (Wald = 4.195, *p* < 0.05).

These findings demonstrate that 60 Gy gamma irradiation not only impacts the growth and survival of F_1_ progeny but also induces sterility traits inherited by F_2_ descendants, further contributing to the population suppression of *P. solenopsis*.

## 3. Discussion

The SIT relies on releasing large numbers of sterile insects to compete with wild populations for mates, effectively reducing the pest population by preventing successful reproduction. This method requires an optimal irradiation period and dose to achieve complete or sub-sterility without compromising the competitiveness of irradiated insects in the field [11]. In this study, male pupae of *P. solenopsis* were selected as irradiation subjects to minimize somatic damage and maintain the quality of sterile adults. Five γ-ray doses (20, 40, 60, 100, and 200 Gy) were tested to identify the optimal age and dose for sterilization based on impacts of growth, reproduction, and mating competitiveness.

The choice of developmental stage for insect irradiation depends on reproductive organ maturity, sensitivity to somatic cell damage, and operational convenience [11]. In general, pupae tend to be less sensitive to irradiation than larvae but more sensitive than adults, while eggs are the most sensitive of all stages [31]. Among adults, females are more radio-sensitive than males [32,33]. Older pupae and newly emerged adults are commonly used. In *P. solenopsis*, male pupae provide an ideal stage due to their distinct developmental process. Our results showed that 5-day-old male pupae exhibited the highest emergence rate and lowest mortality and deformity rates after irradiation, making them the most suitable for γ-ray exposure. Additionally, pupae are easier to handle and transport compared to fragile eggs or active adults, aligning with findings from studies on *Aedes albopictus* Skuse (Diptera: Culicidae) [34], *Bactrocera dorsalis* Hendel (Diptera: Tephritidae) [35], and *Ephestia elutella* Hübner (Lepidoptera: Pyralidae) [31].

Existing research has indicated that the dose for complete sterility in *P. solenopsis* is 200 Gy, and this dose might suffice as a phytosanitary treatment against the solenopsis mealybug [24]. High-dose irradiation often reduces the mating competitiveness of insects in the field [19,36,37]. Sub-sterile doses, which impair reproduction while maintaining competitiveness, are more effective for population suppression and mainly focus on Lepidoptera [13,15,31] and Diptera [19,38]. Our findings demonstrated that a 60 Gy significantly reduced egg production and hatching rates without negatively affecting male longevity or emergence rates. This dose also effectively suppressed offspring populations. When irradiated F_1_ males mated with non-irradiated females, the egg production decreased by 62.48% compared to non-irradiated male-female pairs. Among the eggs laid, the hatching rate was 56%, and the survival rate of the hatched offspring to adulthood was only 42%. Additionally, the proportion of female adults emerging from these offspring was reduced to 50%, significantly lowering the mealybug population. Therefore, this dose is considered an ideal sub-sterile dose for SIT applications.

The mating competitiveness of irradiated males is crucial for SIT success [39]. Our study found that males irradiated at 60 Gy had a competitiveness value of 2.21, indicating superior mating competitiveness compared to unirradiated males. However, the ratio of sterile to wild males affects population-level fitness and SIT efficacy [15,19,40,41]. Future research should explore the overflooding ratio required for effective suppression of *P. solenopsis* wild populations, as well as the timing of releases. For example, releasing sterile males during the early and mid-flush cotton seasons could maximize population suppression by targeting critical reproductive periods.

Sterility induced by irradiation in the F_1_ generation of irradiated male Lepidoptera insects is often inherited by their descendants, a phenomenon well-documented in *Tuta absoluta* Meyrick (Lepidoptera: Gelechiidae) [13], *Spodoptera frugiperda* Smith (Lepidoptera: Noctuidae) [32], *Teia anartoides* Walker (Lepidoptera: Lymantriidae) [42], *Helicoverpa armigera* Hübner (Lepidoptera: Noctuidae) [43], and *Xestia c-nigrum* Linnaeus (Lepidoptera: Noctuidae) [14]. After irradiation with a sub-sterilizing dose, the sterility traits could be passed on to their offspring, resulting in a continuous decrease in the population of F_1_ and F_2_ generations [44]. Similarly, our study showed that the fecundity of unirradiated females mated with F_1_ males from irradiated parental males declined significantly, with sterility levels higher in the F_1_ than the F_0_ generation. This suggests that irradiation-mediated chromosomal damage is transmitted to offspring, further reducing population growth. This phenomenon has been successfully applied in pest control, such as *Trichoplusia ni* Hübner (Lepidoptera: Noctuidae) [45], *Helicoverpa zea* Boddie (Lepidoptera: Noctuidae) [46], and *Thaumatotibia leucotreta* (Meyrick) (Lepidoptera: Tortricidae) [47].

The γ-ray irradiation-based SIT offers a promising, eco-friendly method for managing *P. solenopsis*. However, further research is required to address several key aspects: (1) Behavioral performance: Comparing the behavioral performance of sterile males versus fertile males under choice conditions, including evaluations at different overflooding ratios. (2) Female sexual behavior: Investigating the sexual behavior of females to determine whether they exhibit monogamous or polyandrous mating patterns. (3) Post-mating sperm selection: Examining the potential for post-mating physiological selection of sperm by females. (4) Mass rearing and field application: Developing and optimizing mass-rearing systems to produce large quantities of male pupae for irradiation as well as determining optimal release ratios to maximize field efficacy and assess the long-term impacts of SIT on pest population dynamics. By incorporating findings from this study, SIT could be effectively adapted for regional pest control strategies, reducing reliance on chemical pesticides and supporting sustainable agricultural practices.

## 4. Materials and Methods

### 4.1. Insects

*Phenacoccus solenopsis* used in this study was collected from *Portulaca grandiflora* in the suburbs of Hangzhou, Zhejiang Province, China. The population was maintained on potato plants and sprouted potato tubers under controlled conditions at 27 ± 1 °C, 60% ± 5% relative humidity, and a 16 h light:8 h dark cycle.

### 4.2. Selection of Optimal Day Age for P. solenopsis Sterility

The pupal stage of *P. solenopsis* lasts approximately 5 days, based on laboratory observations. Male pupae aged 1 to 5 days were selected as research subjects to identify the optimal age for sterility. Pupae were collected and sexed following the method described by Zhao [48]. Ten male pupae per replicate were placed in glass tubes (1.5 cm diameter, 8.0 cm height) and subjected to irradiation, with nine replicates for each dose. To ensure the accuracy of the irradiation dose, the irradiation equipment (xN658, 7.77 × 10^14^ Bq) at the Zhejiang Academy of Agricultural Sciences Radiochemical Center is calibrated twice annually by the National Institute of Metrology, China. The calibration procedure involves measuring the total dose at intervals of 5 cm from 0 to 120 cm from the radiation source using silver dichromate dosimeters (U_rel_ = 4%, k = 2) over an accumulated time of >10 h. The dose rate at each point is then calculated by dividing the total dose by the irradiation time.

In this experiment, the selected measurement point was located 40 cm from the radiation source. Radiation doses of 20, 40, 60, 100, and 200 Gy were administered with exposure times of 0.25, 0.5, 0.75, 1.25, and 2.5 h, respectively, at a dose rate of 80 Gy/h. The glass tubes with pupae were centrally positioned at the designated measurement point facing the Caesium-137 source during irradiation. Irradiation was conducted under normal atmospheric conditions, with the glass tubes left unsealed to allow air exchange. Irradiated pupae were transferred individually to plastic tubes (2.3 cm diameter, 9.3 cm height) to observe emergence, with unirradiated pupae serving as controls. Emergent adults were examined for deformities, and rates of deformity, emergence, and adjusted mortality were calculated to identify the optimal day age for sterility.

### 4.3. Preliminary Selection of Optimal Radiation Dose for P. solenopsis Sterility

Successfully emerged F_0_ male adults from irradiated pupae were paired with unirradiated adult females. Each mating pair was placed in a separate cup and given potato leaves for food, which were replaced daily. Their eggs were quantified and collected.

Due to the high deformity rate and adjusted mortality rate of male pupae irradiated with 200 Gy, which failed to meet the requirements of irradiation sterility, no follow-up experiments were conducted. Four irradiation-treated groups (20, 40, 60, and 100 Gy) and a control group (0 Gy) were tested with three replicates of 15 pairs per treatment. The following parameters were recorded: mating rate, oviposition period, number of eggs laid, hatching rate of F_1_ eggs, male adult lifespan, and sex ratio of F_1_ females. The optimal radiation dose for sterility was preliminarily selected based on these observations.

### 4.4. Effect of 60 Gy Gamma Radiation on Mating Competitiveness of F_0_ Males

One hundred male pupae were irradiated at the optimal dose (60 Gy). Successfully emerged males were subjected to three treatments:

- Fifteen unirradiated males (UMs) and fifteen unirradiated females (UFs) (1:0:1);
- Fifteen irradiated males (IMs) and fifteen UFs (0:1:1);
- Ten UMs, ten IMs, and ten UFs (1:1:1).

In each treatment, adults were released into cages (25 cm × 25 cm × 25 cm) for mating. After the males died, the females were transferred to plastic jars (8.5 cm diameters, 10 cm height) containing sprouted potato tubers. Eggs laid by females were collected daily and monitored for fecundity and hatching rate. Five replicates were included for each treatment. Mating competitiveness was calculated using Fried’s equation [49] as follows:

$$Mating\;Competitiveness\;Value\;\left(C\right)=\frac{N}{S}\times \frac{H_{a}-H_{o}}{H_{o}-H_{s}}$$

*H_a_*, hatching rate of eggs of UM × UF; *H_s_*, hatching rate of eggs of IM × UF; *H_o_*, hatching rate of eggs of IM × UM × UF; *N*, number of UMs; *S*, number of IMs.

The variance of *C* was calculated using the method of Hooper and Horton [50].

### 4.5. Effect of 60 Gy Gamma Radiation on Descendants of Irradiated F_0_ Males

Irradiated male adults (IM) from 60 Gy-irradiated pupae were paired with unirradiated female adults (UF). Eggs laid by UF were collected, and F_1_ eggs from unirradiated male adults (UM) and females served as controls. Developmental duration and survival rates were observed for approximately 200 eggs and 60*–*80 first-instar nymphs per replicate, with three replicates per treatment.

To estimate inherited sterility, newly emerged F_1_ adults from IM × UF pairings were mated with unirradiated mealybugs of the opposite sex. Fecundity and the growth status of F_2_ eggs and nymphs were recorded, with at least 200 eggs and 60*–*80 first-instar nymphs observed per replicate, with three replicates per treatment.

### 4.6. Data Analysis

A two-way ANOVA was used to examine the adjusted mortality, pupal emergence rate, and deformity rate of F_0_ males, with different radiation doses and age as factors. Prior to the ANOVA, percentage data were converted by arcsine square root. Effective dose (ED) values for mortality were estimated using Probit analysis. A negative binomial regression was performed for count data by using the generalized linear model (GLM) procedure and a logistic regression for proportional variables. Specifically, the effect of gamma radiation with different doses on the oviposition stage, longevity, and fecundity of F_0_ adults; the effect of different ratios of unirradiated males, irradiated males, and unirradiated females on fecundity; and the effect of optimal radiation dose on F_1_ fecundity were analyzed using a negative binomial regression. The effect of gamma radiation with different doses on the mating rate of F_0_ adults and F_1_ (hatching rate of F_1_ eggs and sex ratio of F_1_ females), the effect of different ratios of unirradiated males, irradiated males and unirradiated females on hatching rate, and the effect of optimal radiation dose on F_2_ (hatching rate of F_2_ eggs, survival rate of F_2_ pre-adult stage, and sex ratio of F_2_ females) were analyzed by a logistic regression. Multiple comparisons of biological parameters of *P. solenopsis* among radiation doses or types of couples were performed using Duncan’s multiple range tests (*p* < 0.05). Independent-sample *t*-tests compared developmental duration and survival rates between treated and control groups.

All analyses were conducted using SPSS 22.0 (SPSS, Chicago, IL, USA), and graphs were generated using GraphPad (v9.0, San Diego, CA, USA). Data represent mean ± standard error.

## 5. Conclusions

We first evaluate the effects of γ-ray irradiation-based SIT on *P. solenopsis*. Five-day-old male pupae were identified as the optimal developmental stage for irradiation, exhibiting a higher tolerance to radiation compared to younger pupae. A γ-ray dose of 60 Gy was determined to be the most effective for inducing sterility in male pupae without compromising their mating competitiveness. These findings establish the scientific basis and feasibility of using γ-ray irradiation for the management of *P. solenopsis*. This approach offers a promising, eco-friendly alternative for regional control of this pest, paving the way for implementing SIT strategies in agricultural pest management programs.

## Figures and Tables

**Table 1:** Summary of two-way analysis of variance (ANOVA) of *Phenacoccus solenopsis* with different radiation doses and ages (SS = sum of squares; MS = mean squares).

Target Variable	Source	Type III SS	df	MS	*F*	*p*
Adjusted mortality	Radiation dose	27,755.311	4	6938.828	1782.452	<0.001
	Age	3105.778	4	776.444	199.454	<0.001
	Radiation dose × Age	2182.929	16	136.433	35.047	<0.001
Emergence rate	Radiation dose	36,313.534	5	7262.707	1131.385	<0.001
	Age	3235.275	4	808.819	125.998	<0.001
	Radiation dose × Age	2139.412	20	106.971	16.664	<0.001
Deformity rate	Radiation dose	24,583.731	5	4916.746	509.003	<0.001
	Age	3703.982	4	925.996	95.863	<0.001
	Radiation dose × Age	4275.637	18	237.535	24.591	<0.001

**Table 2:** Effective dose values of different pupae day age of *Phenacoccus solenopsis* based on mortality.

Pupae Day Age	Number	Slope ± SE	Estimated Doses (95% CI) (Gy)		χ^2^ (*df*)	*p* Values
			ED_50_	ED_99_		
1-day-old	559	2.30 ± 0.39	75.23 (64.99–85.09)	206.54 (160.22–335.86)	2.64 (12)	0.998
2-day-old	644	2.67 ± 0.58	100.96 (85.96–112.52)	241.65 (189.77–427.10)	9.74 (12)	0.639
3-day-old	662	2.33 ± 0.46	121.94 (101.95–137.37)	331.43 (256.82–577.82)	5.16 (12)	0.852
4-day-old	694	1.67 ± 0.32	137.98 (114.62–159.22)	554.53 (385.05–1204.22)	3.81 (12)	0.987
5-day-old	672	1.58 ± 0.35	172.84 (145.51–209.03)	752.34 (466.27–2473.90)	1.33 (12)	1.000

**Table 3:** Mating competitiveness of mealybug at different ratios of sterile (60 Gy) and normal males under laboratory conditions.

Cross Ratio (UM × IM × UF)	Fecundity (Per Female)	%Observed Egg Hatch	Competitiveness Value (*C*)	Variance of *C*
1:0:1	228.84 ± 3.35 a	73.75 ± 1.30 a		
0:1:1	200.68 ± 4.21 b	44.94 ± 1.74 c		
1:1:1	211.53 ± 1.94 b	52.57 ± 1.04 b	2.21	6.34

Within a column, means followed by different letters are significantly different among different types of couples (*p* < 0.05). Figures in the “% observed egg hatch” column represent the egg hatching rates transformed using the arcsine square root transformation; UM, unirradiated male; IM, irradiated male; UF, unirradiated female.

## Data Availability

Data are contained within the article.
